# Author Correction: Ketamine can reduce harmful drinking by pharmacologically rewriting drinking memories

**DOI:** 10.1038/s41467-020-16762-z

**Published:** 2020-06-11

**Authors:** Ravi K. Das, Grace Gale, Katie Walsh, Vanessa E. Hennessy, Georges Iskandar, Luke A. Mordecai, Brigitta Brandner, Merel Kindt, H. Valerie Curran, Sunjeev K. Kamboj

**Affiliations:** 1 Clinical Psychopharmacology Unit, University College London, London, UK; 2 University College Hospital and University College Hospital at Westmoreland Street, London, UK; 3 Pain Management Centre, University College Hospital, London, UK; 4 Experimental Clinical Psychology, University of Amsterdam, Amsterdam, The Netherlands

**Keywords:** Learning and memory, Reward, Addiction, Therapeutics

Correction to: *Nature Communications* 10.1038/s41467-019-13162-w, published online 26 November 2019.

The original version of this article contained several errors as described below.

The significance bar for ‘*p* < 0.004’ was incorrectly placed above the RET + PBO group in Fig. 2b. The correct version places it above the ‘No RET + KET’ group. Figure 3 contained an error, the data points shown in the middle and lower panel had been inadvertently duplicated during the revision process. This has been corrected. The ‘Author contributions’ incorrectly read ‘S.K.K. managed the research and edited the paper’. The correct version states ‘S.K.K. managed the research and edited the paper, helped design the study and secured funding’. The description of dose of ketamine used in the ‘Methods’ incorrectly read ‘350 ng/dl’. The correct version states ‘350 ng/ml’ in place of ‘350 ng/dl’. These errors have been corrected in both the PDF and HTML versions of the article.

The original version of this article contained two errors in the Supplementary Information.

The description of the units of ketamine used in Supplementary information on page 3 and page 4 incorrectly read ‘ng/dl’. The correct version states ‘ng/ml’ in place of ‘ng/dl’. A sentence on page 6 incorrectly read ‘This reflected significant acute increases in hedonic tone from pre-drug to on-drug in the groups receiving ketamine’. The correct version states ‘decreases’ instead of ‘increases’. The HTML has been updated to include a corrected version of the Supplementary Information.

