# Supplementary material for: Ketamine can reduce harmful drinking by pharmacologically rewriting drinking memories
Source: Nat Commun. 2019 Nov 26;10:5187. doi: 10.1038/s41467-019-13162-w (PMC6879579; doi:10.1038/s41467-019-13162-w)
Supplement: Supplementary file 1 — Supplementary Information [file 41467_2019_13162_MOESM1_ESM.pdf]

SUPPLEMENTARY INFORMATION

Das, RK et al: Ketamine can reduce harmful drinking by pharmacologically rewriting drinking memories.

SUPPLEMENTARY METHODS:

Telephone screens were conducted to assess eligibility to take part in the experiment, using the following criteria: Inclusion criteria: Scoring >8 on the Alcohol Use Disorders Identification Test (AUDIT)[1]; Consuming > 40 (men) or >30 (women) UK units/week, primarily drinking beer, non-treatment seeking for AUD or any other psychiatric disorder; drinking ≥ 4 days/week, drinking >3 units on drinking days, ages 18-65, being 'highly motivated to reduce' drinking, BMI >18<35.

Exclusion criteria: Meeting SCID (DSM IV) criteria for physiological alcohol dependence at screening, having a formal diagnosis of alcohol or substance use disorder (AUD/SUD) or any other psychiatric disorder, undergoing or seeking treatment for alcohol use, current use of any centrally active medications, use of recreational drugs (other than tobacco) > 1x/ month, any recreational use of ketamine, major physical health issues contraindicating ketamine, blood pressure > 145/90, allergy to ketamine, pregnancy or breastfeeding.

Note that fulfilment of inclusion/exclusion criteria was based upon telephone screening and self-report. With the exception of alcohol breath test, we were unable to verify other drug use or abstinence via urine test, similarly, with the exception of blood pressure and BMI assessments, existence or absence of medical conditions was dependent upon self-report, since exhaustive lab testing of medical conditions was beyond the scope of the current study.

The sample were largely young-to-middle aged adults (mean age 27.5 ± 8.1 yrs). Despite lacking formal medical diagnoses of AUD nor seeking treatment, the sample had particularly high drinking levels and AUDIT scores (mean 22.13 ± 4.93), considerably exceeding the score of 8 denoting harmful drinking and moderate-high risk of AUD.

Participant numbers were randomized to groups using a code from random.org at the beginning of the study. Participant numbers were then assigned sequentially to participants following completion of *Day 1* testing. Allocation to groups was even, unstratified and truly random. Randomization was performed by SKK, who was not involved in the screening or testing of participants or analysis of the data. Recruitment started on 12/06/2015 and data collection ran until 01/11/2018. Recruitment ended when randomization of the pre-specified number of participants (N =30/group) was completed and when the final follow-up period (9 months) for the final participant had elapsed.

A battery of questionnaire measures was collected on *Day 1/Day 10* to assess state/trait variables of mood, behaviours, attitudes and cognitions related to drinking. These were the Beck Depression Inventory (BDI)[2], Barratt Impulsiveness Scale (BIS)[3], Behavioural Inhibition/Activation Scale (BIS/BAS)[4], Distress Tolerance Scale (DTS)[5], Positive and Negative Affect Scale (PANAS)[6], Alcohol Use Disorders Identification Test (AUDIT)[1], Timeline Follow-Back (TLFB)[7] for alcohol, Alcohol Craving Questionnaire (ACQ-NOW)[8], Stages of Change Readiness and Eagerness Scale (SOCRATES[9]), Comprehensive Effects of Alcohol (CEOA) scale[10], Obsessive Compulsive Drinking Scale (OCDS)[11]. To complement the TLFB and assess perceived changes in drinking, we used single-item Likert scales asking how much participants 'enjoyed drinking', 'actually drank' and had the 'urge to drink' compared to before the intervention day (-2 = much less, 0 = about the same, +2 = much more).

Baseline and post-manipulation (i.e. *Day 1* and *Day 10*) cue reactivity was assessed via 'liking' and 'urge to drink' ratings of a set of beer (N = 7), wine (N = 3) orange juice (N = 4) and soft drink (N=2) cue images, as described previously[12]. The experimenter first opened a bottle of lager (Pilsner Urquell) in front of the participants and poured 150ml into a half-pint glass. This was placed on the table in front of the participants and they were told that they would drink this beer when instructed to by on-screen prompts, but that first they would rate a series of images for *pleasantness* (liking) and their effects on *urge to drink* (wanting) the beer in front of them. All ratings were made verbally on a scale of -5 (extremely unpleasant/ greatly reduces urge) to +5 (extremely pleasant/ greatly increases urge) and noted by the experimenter. Images were 400x400 pixels, presented centrally on a computer screen, in a random order, for 10 seconds each. Following completion of the rating, participants were asked to rate their current *urge to drink* the *in vivo* beer (*anticipatory urge* rating) and how much they

anticipated they would enjoy the beer when they drank it (*anticipated enjoyment*). A series of prompts then appeared, instructing participants to 1) 'pick up' the drink 2) 'prepare to drink' and 3) 'Drink now'. Upon presentation of the 'drink now' prompt, participants drank the entire 150ml beer in a self-paced manner. After consumption of the beer, participants rated how much they actually enjoyed the beer (*actual enjoyment*) and their desire to drink more (*urge to drink more*) on the same -5 to +5 scale. Following this, the glass and any beer-related paraphernalia was removed. Conducting the cue reactivity/alcohol reinforcement task on *Day 1* and *Day 10* both provided a metric of clinically-relevant changes in the hedonic and motivational effects of beer and maximised the expectancy of receiving beer during the *Day 3* reactivation procedure, thus generating a prediction error (PE) when the drink was withheld on *Day 3*.

The alcohol MRM (RET) and Control (No RET) Memory Reactivation Procedures took place on *Day 3* and used sub-sets of stimuli from the cue reactivity /alcohol reinforcement task. For MRM retrieval (*RET* groups), these were four images of beer and for *No RET+KET*, these were four images of orange juice. All participants also rated two 'soft drink' images of cola and coffee. Participants in MRM retrieval conditions were told they would repeat the image rating and beer consumption task from *Day 1*. Again, a beer was opened and 150ml poured into a glass placed in front of participants. They then rated four of the beer cue images (designated 'beer retrieval' images) and the two soft drink images, along with their 'urge to drink' and anticipated enjoyment of the *in vivo* beer. The drinking prompt screens then began, but the final prompt read 'Stop! Do not drink'. This latter procedure aimed to generate negative prediction error (PE)/ surprise, a putative key determinate of memory destabilisation[13,14]. The glass of beer was then removed and participants rated how much they expected what just happened from -5 (completely unexpected) to +5 (completely expected). This 'surprise' rating served as an explicit measure of PE. In the *No RET+KET* condition, the procedure was identical, barring the following exceptions: 1) A 150ml glass of orange juice was poured and given to the participants. They were instructed that they would drink this after rating some images 2) The images rated by the participants were the four orange juice images and two soft drink images from *Day 1*, thus there was no exposure to any beer or alcohol-related cues. The orange juice was still unexpectedly withheld following the rating, equating the groups for PE. On *Day 3*, acute subjective responses to ketamine/placebo infusions were assessed via the Clinician Administered Dissociative States Scales (CADSS)[15], Bodily Symptoms Scale (BSS)[16], Drug Effects Questionnaire (DEQ)[17] and Snaith-Hamilton Pleasure Scale (SHAPS)[18].

Immediately following the MRM reactivation (*RET*) or control (*No RET*) procedures on *Day 3*, participants completed brief distractor tasks. These were the prose-recall (immediate) task from the Rivermead Behavioral Memory Test battery [19], followed by the digit-span forwards and backwards. The purpose of these tasks was to provide a standardised, high-working memory-load distractor from the preceding RET/No RET procedure and ensure that the retrieval truly terminated when intended to. The rationale for these distractors is that participants may otherwise retain the reactivated memory in working memory, ruminating upon the withheld alcohol and introducing unconstrained variability into the length of the retrieval procedure.

All *Day 1/Day 10* procedures were performed in testing laboratories at University College London. Participants provided consent, then completed the AUDIT, OCDS, COEA, TLFB, SOCRATES, BDI, BIS, BIS/BAS, DTS, PANAS, and ACQ-NOW prior to the cue reactivity task. They were reminded of the fasting requirements for the second session. *Day 10* was identical to *Day 1*, with participants receiving £80 payment upon completion. Follow-up assessments were conducted remotely using Qualtrics software and incentivised at £5 per completion.

Day 3 procedures took place on a clinical ward in University College London Hospital (UCLH). Participants completed the baseline (pre- infusion) SHAPS, CADSS, and BSS and were cannulated by the attending anaesthetist. They then completed the appropriate reactivation / non reactivation procedure, followed by distractor tasks before the anaesthetist began the ketamine/placebo infusion.

The dose of ketamine used in the current study was based on extensive piloting work. Our aim was to determine the highest non-anaesthetic dose of ketamine that could be readily tolerated for the 30 minute infusion period. We piloted concentrations of 200, 250, 300 and 350 ng/dl, based upon

consultation with three consultant anaesthetists, with previous experience of administering ketamine in a research setting and on the basis of doses used in by Morgan and colleagues [20]. During piloting the most common side-effect was nausea, which was effectively managed by a standard 10mg dose of domperidone prior to infusion is the actual study. The highest dose was well-tolerated and was therefore selected for use in the full study.

A racemic preparation of ketamine HCl was used for drug infusions in the *KET* groups. This was prepared in physiological saline by the attending study anaesthetist in a manner blind to the participants and experimenters. Placebo was a matched volume of physiological saline prepared for infusion in an identical manner. Participants were cannulated in their non-dominant hand or forearm and infusions were administered by a (Graseby 3400) syringe pump, controlled by a computer running a three compartment pharmacokinetic (Domino) model of ketamine (STANPUMP) to achieve a blood concentration of 350ng/dl within two minutes and maintain this concentration throughout the 30 minute infusion. Physiological monitoring was performed by the anaesthetist throughout and after the infusions. All infusions were performed in a fasted state (2 hours liquid, 6 hours solids) and 1.5-2 hours after consumption of 10mg Domperidone, to prevent any nausea induced by the infusion.

At 15 minutes following infusion onset, participants completed 'on drug' measures: CADSS, SHAPS, BSS, DEQ. The infusion continued until 30 minutes were complete. Post-drug questionnaires data were re-administered 20 minutes following termination of infusion. Participants were kept in the hospital until they were considered fit for discharge by the anaesthetist and were transported home. No unexpected or serious adverse responses were observed in response to the manipulations. N = 2 participants in *RET+PBO* reported feeling nausea and displayed a brief vasovagal response (prior to any drug administration) in response to venepuncture and N = 3 reported nausea in response to ketamine during administration (N=2 *RET+KET*, N = 1). No participants withdrew from the study following manipulation due to adverse events.

SUPPLEMENTARY NOTE 1:

While single-blinding was attempted for the study, the clear subjective effects of ketamine meant true blinding was not possible. Of the 30 participants receiving PBO, N = 3 guessed they had received ketamine and N = 27 guessed they had received PBO. All 60 participants receiving ketamine correctly guessed that they had received ketamine. This manifested in a highly significant chi square test on condition guess: χ_2 (1, N = 90) = 77.143, $p < 0.001$, indicating that participants were largely able to guess their drug conditions.

It was impossible to blind retrieval condition to the experimenters, as they required different drinks to be given to participants. However, no participants reported knowledge of a differential retrieval-specific manipulation, nor rationale for such. Importantly, follow-up data was collected by an experimenter who unaware of participants' drug and retrieval condition and primary data analysis was performed blind, using a numeric code to refer to groups, the matching of which was unknown to the analyst.

All data analysis was performed using IBM SPSS 25 for Windows and R[21]. All data were checked for outliers, normality, homogeneity of variance and sphericity (for repeated-measures with K>2 comparisons). Where homogeneity of variance was violated in one-way ANOVA, *Welch's F* test is reported, denoted with F_W. For non-sphericity, the Greenhouse Geisser correction or multivariate equivalents of terms used were used, depending ε values and according to the recommendations of Stevens[22]. Outliers >3SD from the mean were winsorized to the highest non-outlying score + 1. For TLFB data, 4 participants' scores (RET+KET = 2, RET +PBO = 1, No RET+KET = 1) were winsorized in this way. Group differences on baseline measures were assessed with one-way ANOVA with false discovery rate (FDR [23] correction to constrain *Type I* error for multiple comparisons of unhypothesized group differences. Primary outcome measures of interest were change in reactivity to alcohol cues and naturalistic drinking from baseline to post-manipulation (*Day 10*). These data were analysed using *Group* X *Time* (baseline/post-manipulation) mixed ANOVA. For naturalistic drinking level (unit consumption) data, to assess whether group differences in alcohol consumption existed

post-manipulation after controlling for baseline alcohol consumption, ANCOVA was used, including a factor of *Group* and baseline unit consumption as a covariate. Due to technical error, one participant's (male, *RET+PBO*) data were lost for the post-intervention time point. Remote follow-up data were collected at 2 weeks, 3, 6 and 9 month intervals following *Day 10*. Due to attrition at each follow-up point and inability to verify follow-up self-report, analyses of follow-up data were conducted separately to analyses of data collected in-lab, where full data were available. Note that if participants did not complete a particular follow-up, this did not preclude them from completing subsequent follow-ups. As such, the *Ns* and *dfs* vary between each follow-up time-point. Linear mixed models were used to analyse these data, specifying random intercepts per-participant, *Group* as a fixed effect and allowing random slopes across *Time* (post-manipulation, 2 weeks, 3 months, 6 months, 9 months). Significant k >2 main effects and interactions in omnibus ANOVAs were investigated with planned, multivariate simple effects analyses of the effect of *Time, with* paired tests on marginal means, where appropriate. Variables were only entered as covariates into analyses of primary outcome variables, where they were found to correlate significantly with the dependent variable. All tests are 2-sided.

Participants' '*liking*' and '*urge to drink*' ratings in response to pictorial cue images in the cue reactivity task were analysed using 2 (*Day*: baseline, post-manipulation) x 5 (*Type: Orange juice*, *Soft drink*, *Wine*, *Reactivated beer*, *Non-reactivated beer*) x 3 (Group) ANOVAs. Here '*reactivated beer*' refers specifically to those images used in the MRM retrieval procedure and '*non reactivated*' refers to beer cue images that were not used in the retrieval procedure. For liking ratings, a *Type* [$F(4, 84) = 17.205$, $p= .001$, $\eta_{p2} = .45$] and *Day* [post-manipulation < baseline $F(1, 87) = 11.163$, $p= .001$, $\eta_{p2} = .114$] main effect were found, under a marginally-significant *Day x Group* interaction [$F(2, 87) = 3.159$, $p= .047$, $\eta_{p2} = .068$]. In line with drinking data, the interaction reflected a general reduction in cue image liking in RET+KET [$F(1, 87) = 15.379$, $p < .001$, $\eta_{p2} = .15$] only.

For cue-induced *urge to drink* ratings, main effects of *Day* [post-intervention < baseline $F(1, 87) = 4.789$, $p= .031$, $\eta_{p2} = .52$] and *Type* [$F(4,84) = 55.063$, $p < .001$, $\eta_{p2} = .724$] were found, under a *Day x Type* interaction [$F(4, 84) = 7.656$, $p < .001$, $\eta_{p2} = .267$]. A trend for a *Day x Group* interaction was also observed [$F(2, 87) = 2.509$, $p < .087$, $\eta_{p2} = .055$] . The *Day x Type* interaction represented a reduction in urge to drink in response to reactivated [$F(1, 87) = 16.786$, $p < .001$, $\eta_{p2} = .162$] and non-reactivated [$F(1,87) = 19.024$, $p < .001$, $\eta_{p2} = .179$] beer cues from pre-to-post manipulation, with no such reductions to other cues [all Fs <3.3, *p*s>.071]. In line with liking data, the *Day X Group* trend represented a generalised reduction in urge to drink in *RET+KET* only [$F(1, 87) = 9.231$, $p=.003$, $\eta_{p2} = .096$].

Changes in the reinforcing properties of *in vivo* beer were assessed by a 2 (*Day*: baseline/post-intervention) x 3 (Group) ANOVA. A *Day* main effect [$F(1, 87) = 9.624$, $p=.003$, $\eta_{p2} = .1$] under a *Day X Group* interaction [$F(2, 87) = 6.489$, $p=.007$, $\eta_{p2} = .109$] was found, for participants' *anticipatory urge* to drink the actual beer in front of them. This indicated a significant reduction in urge to drink the *in vivo* beer in *RET+KET* only [$F(1, 87) = 19.703$, $p <.001$, $\eta_{p2} = .185$; other Fs < 0.5, *p*s > .48]. Similarly, having consumed the 150 ml priming dose of the beer, the *urge to drink more beer* reduced significantly from baseline to post-intervention in *RET+KET* only [*Day x Group* interaction $F(2, 87) = 9.267$, $p=.01$, $\eta_{p2} = .1$; *Day* effect in *RET+KET* [$F(1, 87) = 24.46$, $p <.001$, $\eta_{p2} = .219$]. *Anticipated enjoyment* of the beer followed the same pattern [*Day x Group* interaction $F(2, 87) = 8.234$, $p=.001$, $\eta_{p2} = .159$], with a reduction from baseline to post-intervention in *RET +KET* only [$F(1, 87) = 20.273$, $p <.001$, $\eta_{p2} = .189$]. Correspondingly, participants' actual *enjoyment* of the sampled beer reduced from baseline to post-intervention in *RET +KET* only [$F(1, 87) = 8.67$, $p=.004$, $\eta_{p2} = .091$] with no significant reduction in the other two groups [*Day x Group* interaction $F(2, 87) = 3.298$, $p=.042$, $\eta_{p2} = .07$].

On *Day 10*, participants were asked to retrospectively report on self-perceived changes in drinking behaviour since *Day 1* on a five-point scale (+2 = greatly increased, -2 = greatly decreased). Significant *Group* effects from one-way ANOVA were found for *volume of drinking* [$F(2,87) = 3.164$, $p= .047$, $\eta_2 = .07$], *enjoyment of drinking* [$F(2, 87) = 3.929$, $p= .028$, $\eta_2 = .08$] and *general urge to drink* [$F(2,87) = 5.071$, $p= .008$, $\eta_2 = .1$] since *Day 1*. For volume of drinking, this was due to greater reductions in *RET+KET* than *RET+PBO* [$t(59) = 2.366$, $p=0.05$, $r = .29$]. For *enjoyment of drinking*, this was due to

lower enjoyment in *RET+KET* than *No RET+KET* [$t(59) = 2.581$, $p=0.028$, $r = .32$] and for general *urge to drink*, by lower urge to drink in *RET+KET* than *RET+PBO* [$t(59) = 3.183$ $p=0.001$, $r = .38$].

Although our primary interest was in assessing change in drinking levels, we further assessed whether baseline variability in alcohol consumption affected *Group* estimates of post-manipulation drinking levels, via analysis of covariance on *Day 10* unit consumption data, modelling baseline consumption as a covariate. As expected, baseline consumption co-varied significantly with *Day 10* consumption [$F(1, 85) = 85.2215$, $p < .001$ $\eta_{p2} = .501$]. While there was no overall effect of *Group* [$F(2, 85) = 2.346$, $p=.102$, $\eta_{p2} = .052$], planned contrasts on marginal means revealed significantly reduced estimated alcohol consumption in *RET+KET* vs. *RET + PBO* on post-manipulation drinking [mean difference 16.81 units, $t(57) = 2.16$, $p= 0.034$, $r = .28$]. No significant difference between *No RET+KET* and *RET+PBO* was found [mean difference 9.28 units, $t(57) = 1.21$, $p= 0.23$, $r = .16$]. These data further support the primary analysis in demonstrating the greatest intervention effect in *RET+KET*.

Mixed 2 (baseline/post-intervention) x 3 (Group) ANOVAs were used to assess changes in self-report questionnaire measures problematic drinking. Subscales were analysed separately, as they represent dissociable underlying constructs. For motivation to change, assessed by the SOCRATES questionnaire, no significant changes in any group were seen for 'ambivalence' or 'recognition' (Day main effects $F(1, 86) = .061$, $p= .806$, $\eta_{p2} = .001$ and $F(1, 86) = 1.628$, $p=.205$, $\eta_{p2} = .019$, respectively). However *all* groups were taking more action to reduce their drinking, according to the '*taking steps*' subscale, [$F(1, 86) = 17.561$, $p < .001$, $\eta_{p2} = .17$.] On the OCDS, a significant reduction in obsessive thoughts related to drinking was seen from pre-to-post intervention in all groups [*Day* main effect: $F(1,87) = 25.913$, $p < .001$, $\eta_{p2} = .229$]. A highly significant reduction in compulsive behaviours related to drinking was also observed in all groups [*Day* main effect: $F(1,87) = 65.413$, $p < .001$, $\eta_{p2} = .429$]. These reductions did not significantly differ across groups (No *Group X Time* interaction). In the CEOA measure, significant *Day* effects, (representing improvements in adaptive cognitions regarding alcohol consumption from baseline to test) were seen for the subscales *Sociability*: $F(1, 86) = 26.627$, $p < .001$, $\eta_{p2} = .236$, *Liquid Courage:* $F(1, 86) = 5.215$, $p= .025$, $\eta_{p2} = .057$], *Cognitive Impairment*: $F(1, 86) = 6.603$, $p= .012$, $\eta_{p2} = .071$, *Risk of Aggression*: $F(1, 86) = 14.087$, $p < .001$, $\eta_{p2} = .141$]. No *Group* effects or interactions were observed for any of the subscales.

Depression levels as assessed by the BDI did not differ between groups at baseline (see *Table 1* in main text). However, there was a significant reduction in depression levels between baseline and test in *all* groups [Day main effect: $F(1, 86) = 18.423$, $p < .001$, $\eta_{p2} = .175$]. However, despite the putative anti-depressive effects of ketamine, no effects of *Group* nor interactions were observed, presumably due to the relatively low baseline levels of depression. No significant changes were observed in positive affect as assessed by the PANAS, however a significant *Day X Group* effect emerged for negative affect, [$F(2, 87) = 3.427$, $p= .037$, $\eta_{p2} = .073$] driven by a significant *reduction* in negative affect from baseline to test in *RET+KET* only [$F(1, 87) = 9.106$, $p= .003$, $\eta_{p2} = .095$]. This latter finding is in line with previous data showing improvements of negative mood symptomatology following ketamine, although this has not previously been shown in a (relatively) healthy population and it remains unclear why such an effect was not observed in *No RET+KET*. It is possible that the reduction in negative affect was a secondary benefit to the reductions in drinking observed in *RET+KET*. To assess whether these changes in PANAS-rated negative affect (NA) could explain variance in drinking outcomes, change scores in NA were calculated as *Day 10* score - *Day 1* score and correlations between these change scores and total unit consumption were assessed at each time point. No significant correlations were found between NA change and drinking levels at any time point across the sample as a whole (all $r < \pm .068$, all $p > .53$). Nor were any associations between NA and drinking observed when looking at *RET+KET* specifically (all $r < \pm .182$, all $p > 0.34$). As such, change in negative affect was unlikely to underlie the observed changes in drinking.

Analysis of *Day 3* state measures of mood in response to ketamine measures were assessed with *Time* (Pre-infusion, during infusion, post-infusion) X *Group* ANOVAs. A *Time X Group* interaction emerged on the SHAPS $F(4,172) = 4$, $p= .004$, $\eta_{p2} = .085$]. This reflected significant acute increases in hedonic tone from pre-drug to on-drug in the groups receiving ketamine, with a subsequent return to baseline

levels following cessation of infusion [*Time* simple effects: *RET+KET*: F(2, 85) = 10.073, $p < .001$, η_{p2} = .192, *No RET+KET*: F(2, 85) = 19.229, $p < .001$, η_{p2} = .312]. No changes across time points were seen in the group receiving placebo F(2, 85) = .385, p= .682 η_{p2} = .009].

The same pattern was observed for state levels of dissociation as assessed by the CADSS [*Time X Group* effect F(4, 172) = 35.281, $p < .001$, η_{p2} = .451]. Both *RET+KET* [*Time* simple effect F (2, 85) = 107.194, $p < .001$, η_{p2} = .716] and *No RET+KET* [*Time* simple effect F (2, 85) = 55.083, $p < .001$, η_{p2} = .564] showed large transient increases in dissociation on-drug, followed by large decreases post-infusion, with no change observed in RET+PBO [F (2, 85) = .342, p= .711, η_{p2} = .008]. These data are shown in *Supplementary Figure 1*. Further analysis within *RET+KET* and *No RET+KET* groups only revealed a *Group X Time* interaction [F(2, 114) = 3.875, p=.024, η_{p2} = .064]. Participants in *RET+KET* displayed higher mean dissociation when on-drug *than No RET+KET* [F(1, 57) = 4. 584, p= .037, η_{p2} = .074]. No correlations were found between the on-drug CADSS and cue reactivity or naturalistic drinking post-manipulation, however, suggesting that dissociation was unlikely to account for the differential effects on these measures. Indeed, exploratory analysis of drinking data showed no covariate effect of CADSS [F(1,84) = .001, p=.973, η_{p2} < .001], nor interaction with Group [F(1,84)=0.131, p=.719, η_{p2} = .002], confirming that this was not the case.

On the Drug Effects Questionnaire VAS measure, highly significant *Group* effects were found for *Feeling a Drug Effect* [Fw(2, 44.258) = 277.913, $p < .001$, η_2 = .806]; *Are you high?* [Fw (2, 39.306)=210.534, $p < .001$, η_2 = .732]; *Disliking of any effects* [Fw(2, 51.317) = 11.802, $p < .001$, η_2 = .178] and *Liking of any effects* [Fw(2, 54.659) = 24.978, p< .001, η_2 = .351]. In all cases this group effect represented higher ratings in the groups receiving ketamine than placebo (all *p*s < .001), with non-significant differences between the two ketamine groups (all *p*s>.2). Satiety was evidenced by a lack of group effect for *Wanting more of the drug* Fw (2, 55.718) = 277.913, p=.138, n_2 = .038.

To assess whether non-amnestic mechanisms, such as state dependency or counterconditioning were likely to be responsible for ketamine's observed effects, we correlated on-drug measures (DEQ) with responses to alcohol (cue reactivity) post-intervention and naturalistic alcohol consumption post-intervention in participants who received ketamine. Item 2 on the DEQ ('*Are you high right now?*') was predictive of post-intervention alcohol consumption *r(58)*= -.339, p=.009 and urge to drink *in vivo* beer *r(58)* =-.34, p=.009. This indicated that a stronger drug effect appeared to lead to greater reductions in drinking and urge to drink beer. However, liking/disliking of ketamine's effects and dissociation were not associated with drinking outcomes, suggesting updating of affective associations surrounding alcohol was unlikely to be responsible for the observed effects.

SUPPLEMENTARY DISCUSSION:

Reviewers highlighted the apparent disparity between the high mean AUDIT scores and sub-threshold SCID scores among the sample. This was driven by differences in the focus of questions in the respective screening tools. Participants scored particularly highly on AUDIT items pertaining to heaviness and frequency of drinking and bingeing (participants frequently scored 9-12 points on AUDIT items 1 to 3 alone). Indeed, as confirmed by the TLFB data, their consumption was generally extremely high. They further scored highly on items assessing guilt/remorse, blackouts and injuries during drinking, very likely to be the result of isolated, heavy binge episodes. However, despite such drinking patterns, their general physical symptomatology (withdrawal, drinking despite problems), inability to complete daily required tasks (neglect of activities) and distress caused by drinking were not particularly high. Furthermore, they virtually never drank in the morning. The SCID is highly skewed to the latter measures of impairment and physical symptomatology in determining AUD. The sample's concern and distress caused by their drinking had never reached a sufficient level to seek treatment for AUD, since such behaviour would have exempted them from participation. Exclusion of participants at screening based on their SCID score further contributed to the disparity in the two measures in the current sample. There is no shortage of drinkers such as these in the UK; i.e. whose *consumption* levels would meet those of clinical criteria, but within their sociocultural milieu, do not see themselves as 'alcoholic', nor find their drinking overly impacts upon their daily function. This

raises important questions about the impact of culture-specific normative expectations upon the validity of different screening/diagnostic tools, the utility of universal cut-offs, what exactly is being assessed by the AUDIT and the SCID and how they are answered. These important issues require further attention in future research.

SUPPLEMENTARY FIGURES

Supplementary Figure 1: Acute dissociative (**A**) and hedonic (**B**) effects of ketamine as assessed by the CADSS and SHAPS. Both *RET+KET* and *No RET+KET* showed significant quadratic responses in these measures and did not differ significantly, whereas *RET+PBO* showed no significant change across the time points. Significance levels represent between-group differences from corrected post-hoc tests on Group. ** = *RET+ KET* & *No RET+KET* > *RET+PBO* $p < 0.01$. Points represent mean ± SEM.

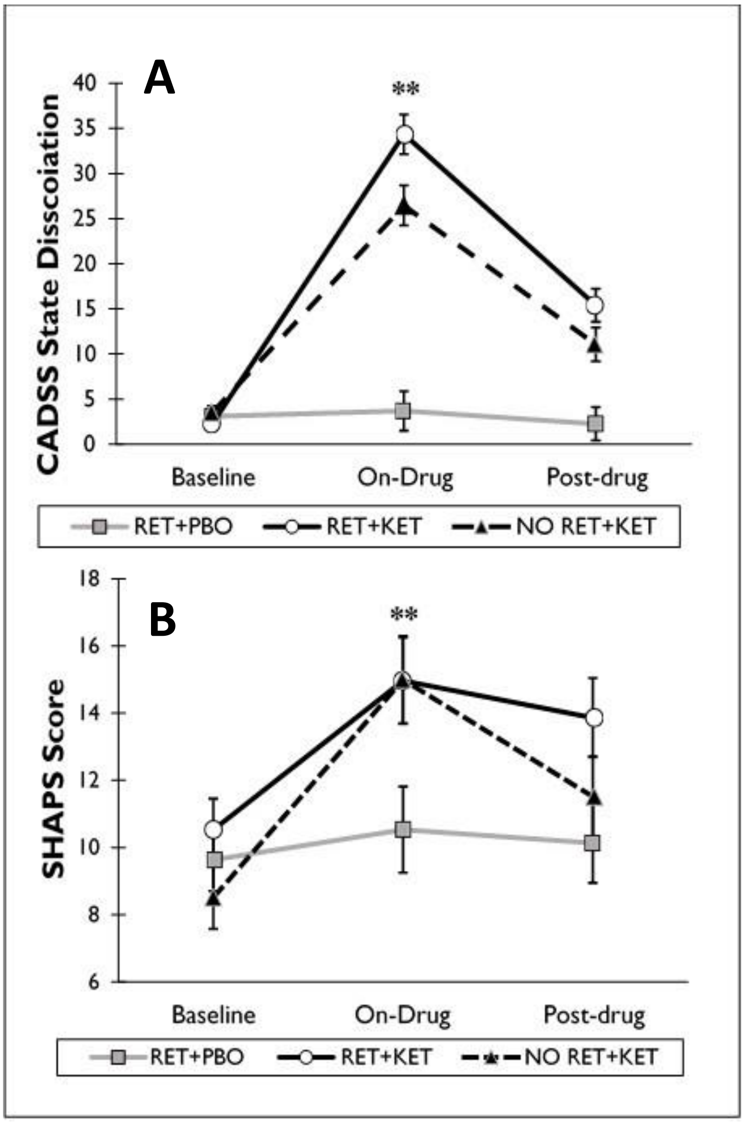

Supplementary References

1. Saunders, J. B., Aasland, O. G., Babor, T. F., De La Fuente, J. R. & Grant, M. Development of the Alcohol Use Disorders Identification Test (AUDIT): WHO Collaborative Project on Early Detection of Persons with Harmful Alcohol Consumption-II. *Addiction* **88**, 791–804 (1993).
2. Beck, A. T., Steer, R. A. & Carbin, M. G. Psychometric properties of the Beck Depression Inventory: Twenty-five years of evaluation. *Clin. Psychol. Rev.* **8**, 77–100 (1988).
3. Patton, J. H. & Stanford, M. S. Factor structure of the Barratt impulsiveness scale. *J. Clin. Psychol.* **51**, 768–774 (1995).
4. Carver, C. S. & White, T. L. Behavioral inhibition, behavioral activation, and affective responses to impending reward and punishment: the BIS/BAS scales. *J. Pers. Soc. Psychol.* **67**, 319 (1994).
5. Simons, J. S. & Gaher, R. M. The distress tolerance scale: Development and validation of a self-report measure. *Motivation and Emotion* (2005). doi:10.1007/s11031-005-7955-3
6. Watson, D., Clark, L. A. & Tellegen, A. Development and validation of brief measures of positive and negative affect: The PANAS scales. *J. Pers. Soc. Psychol.* **54**, 1063–1070 (1988).
7. Sobell, L. C. & Sobell, M. B. Timeline follow-back. in *Measuring alcohol consumption* 41–72 (Springer, 1992).
8. Singleton, E. G., Henningfield, J. E. & Tiffany, S. T. Alcohol craving questionnaire: ACQ-Now: background and administration manual. *Balt. NIDA Addict. Res. Cent.* (1994).
9. Miller, W. R. & Tonigan, J. S. Assessing drinkers' motivation for change: The Stages of Change Readiness and Treatment Eagerness Scale (SOCRATES). *Psychol. Addict. Behav.* **10**, 81–89 (1996).
10. Fromme, K., Stroot, E. A. & Kaplan, D. Comprehensive effects of alcohol: Development and psychometric assessment of a new expectancy questionnaire. *Psychol. Assess.* **5**, 19–26 (1993).
11. Anton, R. F., Moak, D. H. & Latham, P. The Obsessive Compulsive Drinking Scale: A Self-Rated Instrument for the Quantification of Thoughts about Alcohol and Drinking Behavior. *Alcohol. Clin. Exp. Res.* (1995). doi:10.1111/j.1530-0277.1995.tb01475.x
12. Das, R. K., Gale, G., Hennessy, V. & Kamboj, S. K. A Prediction Error-driven Retrieval Procedure for Destabilizing and Rewriting Maladaptive Reward Memories in Hazardous Drinkers. *J. Vis. Exp.* e56097–e56097 (2018). doi:10.3791/56097
13. Das, R. K., Walsh, K., Hannaford, J., Lazzarino, A. I. & Kamboj, S. K. Nitrous oxide may interfere with the reconsolidation of drinking memories in hazardous drinkers in a prediction-error-dependent manner. *Eur. Neuropsychopharmacol.* **28**, 828–840 (2018).
14. Sevenster, D., Beckers, T. & Kindt, M. Prediction error demarcates the transition from retrieval, to reconsolidation, to new learning. *Learn. Mem.* **21**, 580–584 (2014).
15. Bremner, J. D. *et al.* Measurement of dissociative states with the clinician-administered dissociative states scale (CADSS). *J. Trauma. Stress* **11**, 125–136 (1998).
16. Bond, A. & Lader, M. The use of analogue scales in rating subjective feelings. *Br. J. Med. Psychol.* **47**, 211–218 (1974).
17. Morean, M. E. *et al.* The drug effects questionnaire: psychometric support across three drug types. *Psychopharmacology (Berl).* **227**, 177–92 (2013).
18. Snaith, R. P. *et al.* A scale for the assessment of hedonic tone. The Snaith-Hamilton Pleasure Scale. *Br. J. Psychiatry* (1995). doi:10.1192/bjp.167.1.99

19. Wilson, B. A., Cockburn, J. & Baddeley, A. D. *The Rivermead behavioural memory test*. (Thames Valley Test Company, 1991).

20. Morgan, C. J. A., Mofeez, A., Brandner, B., Bromley, L. & Curran, H. V. Acute Effects of Ketamine on Memory Systems and Psychotic Symptoms in Healthy Volunteers. *Neuropsychopharmacology* **29**, 208–218 (2004).

21. R Development Core Team. R: A language and environment for statistical computing. R Foundation for Statistical Computing, Vienna, Austria. URL http://www.R-project.org/. *R Found. Stat. Comput. Vienna, Austria.* (2015). doi:10.1007/978-3-540-74686-7

22. Stevens, J. P. *Applied multivariate statistics for the social sciences*. (Routledge, 2012).

23. Benjamini, Y. & Hochberg, Y. Controlling the false discovery rate: a practical and powerful approach to multiple testing. *J. R. Stat. Soc. Ser. B* 289–300 (1995).
